# Pain Sensitisation in Women with Active Rheumatoid Arthritis: A Comparative Cross-Sectional Study

**DOI:** 10.1155/2015/434109

**Published:** 2015-07-21

**Authors:** Nora Vladimirova, Anders Jespersen, Else Marie Bartels, Anton W. Christensen, Henning Bliddal, Bente Danneskiold-Samsøe

**Affiliations:** ^1^The Parker Institute, Department of Rheumatology, Copenhagen University Hospital, Bispebjerg and Frederiksberg, 2000 Frederiksberg, Denmark; ^2^The Health Science Faculty, University of Copenhagen, 2200 Copenhagen N, Denmark

## Abstract

*Objectives*. In some rheumatoid arthritis (RA) patients, joint pain persists without signs of inflammation. This indicates that central pain sensitisation may play a role in the generation of chronic pain in a subgroup of RA. Our aim was to assess the degree of peripheral and central pain sensitisation in women with active RA compared to healthy controls (HC). *Methods*. 38 women with active RA (DAS28 > 2.6) and 38 female HC were included in, and completed, the study. Exclusion criteria were polyneuropathy, pregnancy, and no Danish language. Cuff Pressure Algometry measurements were carried out on the dominant lower leg. Pain threshold, pain tolerance, and pain sensitivity during tonic painful stimulation were recorded. *Results*. Women with active RA had significantly lower pain threshold (*p* < 0.01) and pain tolerance (*p* < 0.01) than HC. The mean temporal summation- (TS-) index in RA patients was 0.98 (SEM: 0.09) and 0.71 (SEM: 0.04) in HC (*p* < 0.01). *Conclusion*. Patients with active RA showed decreased pressure-pain threshold compared to HC. In addition, temporal summation of pressure-pain was increased, indicating central pain sensitization, at least in some patients. Defining this subgroup of patients may be of importance when considering treatment strategies.

## 1. Introduction

Rheumatoid arthritis (RA) is a chronic inflammatory disease causing synovial joint destruction, disability, and pain [1]. Despite better disease control, many RA patients rate their pain as a major burden, affecting their quality of life [2]. Although physicians often assume that inflammation is the main determinant of pain intensity, more than one in 10 of the patients in DAS28 remission [3] still report clinically significant pain levels [4]. The fact that pain persists despite absence of signs of inflammation may therefore indicate that other factors in the pain processing, for example, sensitisation, either peripherally or centrally, play an important role in the generation of pain in RA [5].

Central pain sensitisation is defined as “an increased responsiveness of nociceptive neurons in the central nervous system to their normal or subthreshold afferent input” [6]. Clinically, this results in hyperalgesia and allodynia and receptive field expansion [7]. It is plausible that subgroups of RA patients experience alterations in their central pain modulation which then becomes a primary cause of pain, possibly outlasting the inflammatory activity. In this subgroup, central pain sensitisation may contribute significantly to high tender joint counts and self-reporting of pain, causing overestimation of disease activity [8]. In RA patients, concomitant fibromyalgia has been found to be around 13–17% [9].

Pain in RA is usually assessed either by self-reported questionnaires or by simple palpation of the joints. Computerized Cuff Pressure Algometry (CPA) is a more precise method for quantitative pain testing which primarily assesses sensitivity of muscle and deep tissue. CPA is less influenced by inter- and intraexaminer bias than handheld pressure algometry [10]. CPA is used to assess different aspects of pain in various rheumatic conditions [11–13]. Pain assessed by CPA has in a group of fibromyalgia shown to correlate to number of tender points, thereby creating a new approach to pain assessment in chronic pain patients [14].

Based on the hypothesis that some RA patients with a high DAS28 suffer from central pain sensitisation, our aim was to use CPA to study pain properties in patients with active RA. The main outcomes of interest were pain threshold and pain tolerance, indicating the degree of peripheral pain sensitivity and degree of temporal summation (i.e., central pain sensitisation) assessed by tonic painful cuff pressure stimulation [13].

## 2. Methods

### 2.1. Study Type and Ethics

This clinical study was cross-sectional and noninterventional carried out according to the Helsinki Declaration and accepted by the Capital Region of Denmark's Ethical Committee (KF01-058/02). All assessments were performed the same day and in the same order by the same persons. All participants gave informed written consent to participate.

### 2.2. Participants

Patients were recruited from the Rheumatology Department, Bispebjerg and Frederiksberg Hospital, Denmark, from June 2011 to September 2012, and were screened according to the following inclusion criteria:

- age ≥ 18;
- RA diagnosed by a rheumatologist according to the ACR criteria [1];
- active RA according to the EULAR definition, DAS28 ≥ 2.6 [3].

**Figure 1:**
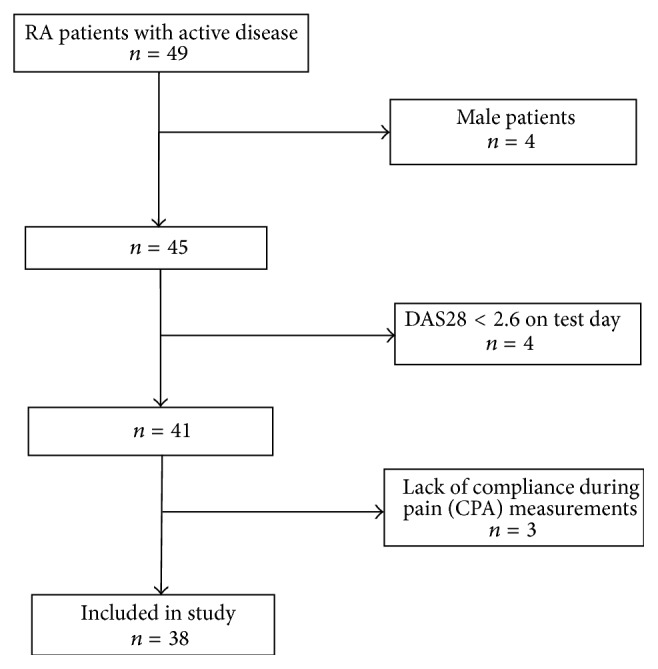
Flow diagram of the selection process of the RA patients. 49 eligible patients were included. Due to gender bias in the control group, the male RA patients were excluded after protocol. Patients not fulfilling the activity criteria, according to DAS28 on the test day, were also excluded. During measurements, 3 patients were excluded due to lack of compliance. The result was enrollment of 38 female patients with active rheumatoid arthritis in the study.

Exclusion criteria were polyneuropathy, pregnancy, and patients not able to understand Danish or to follow instructions.  Figure 1 shows the selection process of the patients enrolled in this study. Healthy controls (HC) were assessed at the clinic at an earlier study to provide data on healthy subjects, and the data was taken directly from this study [13]. The control subjects were all assessed at entry to be healthy, and the protocol for the pain measurements was the same as in the present study.

### 2.3. General Clinical Examinations

The following parameters were measured: A 28-joint count [3], plasma C-reactive protein (CRP) (measured at inclusion in heparin plasma with immunoturbidimetric absorption photometry (Roche/Hitachi cobas-C systems, Roche Diagnostics GmbH, D-68298 Mannheim), with a value ≤10 mg/L being considered normal concentration, and a detection limit of 0.3 mg/L), DAS28, Disease Activity Score in 28 joints based on CRP [3], and manual 18-tender points count [15]. Patient-reported outcomes were registered via computerized Health Assessment Questionnaire [16].

### 2.4. Cuff Pressure Algometry

**Figure 2:**
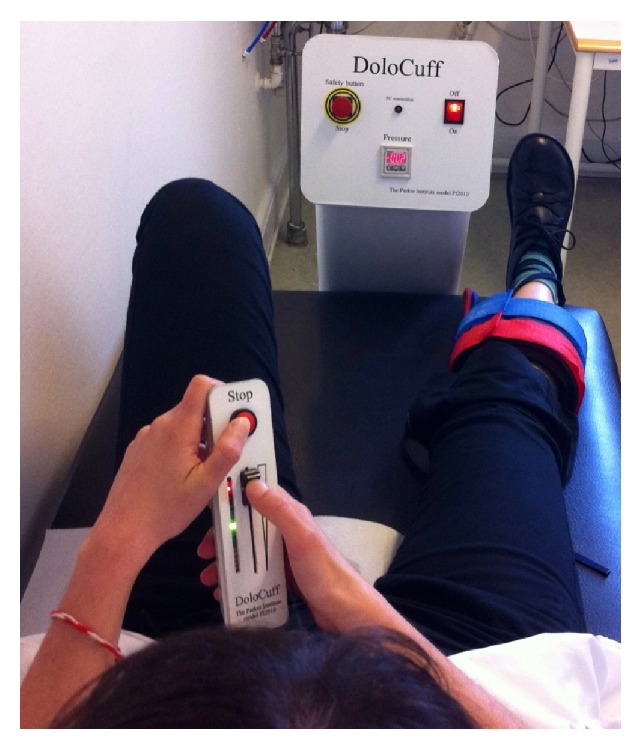
Cuff Pressure Algometry (CPA). The device consisted of a 61 cm tourniquet cuff, a computer-controlled air compressor, and an electronic 10 cm visual analogue scale (VAS). The cuff was placed on the widest part of the lower leg (not in relation to the joints). The measurements were carried out on the subject's dominant side.

The device consisted of a 61 cm tourniquet cuff (placed on the widest part of the lower leg on subject's dominant side), a computer-controlled air compressor, and an electronic 10 cm visual analogue scale (VAS) (DoloCuff, Unique Electronics, Denmark) (Figure 2). The subject was instructed to rate the pain intensity continuously on the VAS, from the first sensation of pain after start of inflation and to press the pressure-release button at “the pain intensity strong enough to make one feel like interrupting or stopping it.” Based on a mean of three consecutive recordings the following variables were determined: (1) pain threshold (unit: kPa), defined as the moment of transition between strong and painful pressure (the first time the VAS exceeds 0) and (2) pain tolerance (unit: kPa), the pressure value at the termination of pressure inflation. Following this, degree of temporal summation was determined according to a predefined protocol [12, 13]. The cuff was filled to obtain a pain intensity based on the average pressure-pain threshold and pressure tolerance of the individual subject. This individualized stimulation was maintained for 10 minutes, while the subject continuously reported pain intensity on the electronic VAS. Based on highest VAS-pain rating, VAS-pain rating at end of stimulation, and Effective Stimulation Time, an index (TS-index) describing degree of temporal summation was calculated according to the following formula:

(1) TS-index=log⁡VASendVASmax∗10TIMEstimulation∗VASend

(see [13]). The TS-index increases with increasing degrees of temporal summation and expresses central pain sensitisation. VAS is measured on the 0–10 scale, and time is measured in minutes.

### 2.5. Statistical Methods

For data analyses, SAS software (v. 9.1.3 Service Pack 4; SAS Institute Inc., Cary, NC, USA) was applied. In order to evaluate the empirical distributions of the continuous outcomes, we tested for normal distribution looking at skewness/SE_skew_ and kurtosis/SE_kurt_.

The PROC MEANS statement was used for summarizing the data. T-TEST procedure was used to compare the two groups, analysed as two independent samples. The CORR procedure, based on Spearman's rank-order correlation, was used as a nonparametric measure of association between various variables.

## 3. Results

The median age of patients was 56 years (IQR 46–69) and 39 (IQR 32–44) of the controls. Characteristics of RA patients are shown in Table 1.

### 3.1. Cuff Algometry Data

Data from Cuff algometry measurements are shown in Table 2.

### 3.2. Clinical Features of the Patients

All RA patients had active disease at the time of assessment, with median DAS-28CRP of 4.3 (IQR 3.6–5.0). The median number of swollen joints was 5 (IQR 3–11) and the number of tender joints was 7 (IQR 4–13). The disease duration varied from 4 to 444 months with a median of 33 months.

### 3.3. Pressure-Pain Thresholds and Tolerance

The mean pressure-pain threshold and pressure-pain tolerance within the RA group were significantly decreased (*p* < 0.01) compared with the healthy controls (Table 1).

### 3.4. Temporal Summation of Pain (TS-Index)

The maximal value for the TS-index was 1.09 in HC; 11 out of 38 (29%) in the RA group had a TS-index above 1.09, with a maximum of 2.46.

**Figure 3:**
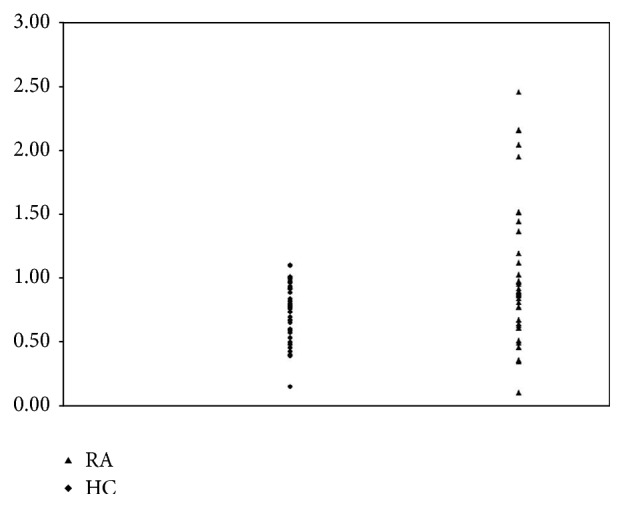
Scatter plot of the TS-index for the RA patients and HC. There is a statistically significant difference in TS-index between the two groups with TS-index significantly higher in RA than in HC, (*p* < 0.01).

The TS-index was significantly higher in RA (0.98, SEM: 0.09) than in HC (0.71, SEM: 0.04) (*p* < 0.01) (Figure 3).

### 3.5. Secondary Analysis

There was no correlation between TS-index and age (HC: *r*_*s*_ = −0.003; *p* = 0.99; RA: *r*_*s*_ = −0.122; *p* = 0.46), disease duration (RA: *r*_*s*_ = −0.281; *p* = 0.09), number of tender points (RA: *r*_*s*_ = 0.169; *p* = 0.31), tender joint count (RA: *r*_*s*_ = 0.269; *p* = 0.10), and swollen joints count (RA: *r*_*s*_ = 0.147; *p* = 0.38), or DAS28 (RA: *r*_*s*_ = 0.281; *p* = 0.09).

## 4. Discussion

Earlier studies have reported concomitant fibromyalgia in a subpopulation of RA patients [9, 17, 18], but this is the first study investigating temporal summation as an indicator of central pain sensitization in RA patients with active disease according to having a DAS28 > 2.6. The study demonstrated that women with active RA (DAS28 > 2.6) showed a significantly decreased pressure-pain thresholds and tolerance on the muscles of the lower leg when compared to HC, indicating a higher degree of peripheral pain sensitivity [19]. Furthermore, temporal summation of pain characterized by the TS-index was significantly higher in RA patients compared with HC. These findings confirm that, as a group, RA patients suffer from nonarticular pain hypersensitivity and facilitated temporal summation, indicating central pain sensitisation [7].

In addition, post hoc analyses indicated that the degree of temporal summation was more pronounced in some of the RA patients, where 29% had a TS-index above the upper limit of that of HC. This suggests that degree of central sensitisation is more pronounced in a subgroup of RA patients [20].

Computerized Cuff Pressure Algometry has previously been applied in other chronic rheumatic pain conditions [12, 13, 21]. There are no previous reports concerning temporal summation assessed by CPA among RA patients but the present results suggest that CPA may be of value in the identification of RA patients with abnormal pain sensitisation.

In RA, pain is the major determinant of patient global assessment of disease activity [22]. In subjects with increased central pain sensitisation this may lead to increased pain reports and overestimates of disease activity [4, 8]. This would also in part explain the significant proportion of RA patients who do not respond to treatment with biologics, since these drugs target proinflammatory pathways [23]. This study indicates that degree of central sensitisation should be evaluated when planning a strategy for medical therapy of the individual RA patient. Interventions against such pain mechanisms may be supplementary, or even alternative to the interventions aimed at decreasing inflammation [24].

### 4.1. Limitations

Due to limited sample size, subgroup analyses were not possible. Our results, however, point towards a necessity of a large scale study assessing the relationship between treatment response and underlying pain mechanisms. Since this study was on women only, we cannot generalize our results to the pain condition in men with RA.

## 5. Conclusion

In patients with active RA, signs of central pain sensitisation were found as indicated by a higher TS-index compared to the group of healthy controls. This indicates that assessment of pain and the underlying mechanisms may be of importance when considering treatment strategies in the individual subjects with RA.

## Figures and Tables

**Table 1:** Patient characteristics and medication for the 38 participating rheumatoid arthritis patients.

Characteristics	Median (interquartile range)
Age, years	56 (46, 69)
Disease duration, months	33 (29, 45)
IgM RF seropositive, number (%)	23 (61)
Anti CCP-positive, number (%) (*n* = 36)	21 (58)
Bone erosions on X-Ray, number (%) (*n* = 37)	12 (32)
Clinical assessment of disease activity	
DAS28	4.3 (3.6, 5.0)
Tender joints	7 (4, 13)
Swollen joints	5 (3, 11)
CRP mg/L	5.5 (0.5, 14)
VAS Global	50 (29, 64)
HAQ-score (*n* = 37)	0.88 (0.63, 1.75)
Tender points	14 (8, 18)

Medication	Numbers (% of total participating patient group)

Patients on DMARDs	34 (89)
Patients on DMARDs + biologics	14 (37)
Concomitant per oral prednisolone	9 (24)
Treatment with 80 mg i.m/i.art steroid within the last 4 weeks	5 (13)

RF: rheumatoid factor, anti-CCP: anticyclic citrullinated peptide, DAS 28: Disease Activity Score, VAS: visual analog scale, VAS Global: patient global assessment of disease severity as measured on a visual analog scale, DMARDs: disease-modifying antirheumatic drugs, i.m/i.art: intramuscular or intra-articular steroid injections.

**Table 2:** Cuff algometry parameters of patients and healthy control subjects.

Cuff algometry parameters	RA patients *n* = 38 mean (SEM)	Healthy controls *n* = 38 mean (SEM)	*p* value
Pain detection threshold (kPa)	16.4 (1.2)	24.9 (2.0)	<0.01
Pain tolerance threshold (kPa)	40.5 (2.5)	55.9 (3.1)	<0.01
TS-index	0.98 (0.09)	0.71 (0.04)	<0.01

A regression analysis showed that there was no relationship between age and the measured pain parameters in the healthy control and the patient group.

The TS-index data was borderline to follow normal distribution with skewness/SE_skew _being just at the limits and kurtosis/SE_kurt_ being well inside the limits of −1.96 and 1.96.

## References

[1] Aletaha D., Neogi T., Silman A. J. (2010). 2010 Rheumatoid arthritis classification criteria: an American College of Rheumatology/European League Against Rheumatism collaborative initiative. *Arthritis & Rheumatism*.

[2] Heiberg T., Finset A., Uhlig T., Kvien T. K. (2005). Seven year changes in health status and priorities for improvement of health in patients with rheumatoid arthritis. *Annals of the Rheumatic Diseases*.

[3] Felson D. T., Smolen J. S., Wells G. (2011). American college of rheumatology/European league against rheumatism provisional definition of remission in rheumatoid arthritis for clinical trials. *Arthritis and Rheumatism*.

[4] Lee Y. C., Cui J., Lu B. (2011). Pain persists in DAS28 rheumatoid arthritis remission but not in ACR/EULAR remission: a longitudinal observational study. *Arthritis Research and Therapy*.

[5] Lee Y. C., Nassikas N. J., Clauw D. J. (2011). The role of the central nervous system in the generation and maintenance of chronic pain in rheumatoid arthritis, osteoarthritis and fibromyalgia. *Arthritis Research and Therapy*.

[6] Loeser J. D., Treede R.-D. (2008). The Kyoto protocol of IASP basic pain terminology. *Pain*.

[7] Graven-Nielsen T., Arendt-Nielsen L. (2010). Assessment of mechanisms in localized and widespread musculoskeletal pain. *Nature Reviews Rheumatology*.

[8] Toms J. A. N., Soukup T., Bradna P., Hrncir Z. (2010). Disease activity composite indices in patients with rheumatoid arthritis and concomitant fibromyalgia. *Journal of Rheumatology*.

[9] Ranzolin A., Brenol J. C. T., Bredemeier M. (2009). Association of concomitant fibromyalgia with worse disease activity score in 28 joints, health assessment questionnaire, and short form 36 scores in patients with rheumatoid arthritis. *Arthritis Care & Research*.

[10] Polianskis R., Graven-Nielsen T., Arendt-Nielsen L. (2001). Computer-controlled pneumatic pressure algometry—a new technique for quantitative sensory testing. *European Journal of Pain*.

[11] Graven-Nielsen T., Wodehouse T., Langford R. M., Arendt-Nielsen L., Kidd B. L. (2012). Normalization of widespread hyperesthesia and facilitated spatial summation of deep-tissue pain in knee osteoarthritis patients after knee replacement. *Arthritis and Rheumatism*.

[12] Jespersen A., Dreyer L., Kendall S. (2007). Computerized cuff pressure algometry: a new method to assess deep-tissue hypersensitivity in fibromyalgia. *Pain*.

[13] Jespersen A., Amris K., Graven-Nielsen T. (2013). Assessment of pressure-pain thresholds and central sensitization of pain in lateral epicondylalgia. *Pain Medicine*.

[14] Amris K., Jespersen A., Bliddal H. (2010). Self-reported somatosensory symptoms of neuropathic pain in fibromyalgia and chronic widespread pain correlate with tender point count and pressure-pain thresholds. *Pain*.

[15] Wolfe F., Smythe H. A., Yunus M. B. (1990). The american college of rheumatology 1990 criteria for the classification of fibromyalgia. Report of the Multicenter Criteria Committee. *Arthritis and Rheumatism*.

[16] Thorsen H., Hansen T. M., McKenna S. P., Sørensen S. F., Whalley D. (2001). Adaptation into Danish of the stanford health assessment questionnaire (HAQ) and the rheumatoid arthritis quality of life scale (RAQoL). *Scandinavian Journal of Rheumatology*.

[17] Wolfe F., Michaud K. (2004). Severe rheumatoid arthritis (RA), worse outcomes, comorbid illness, and sociodemographic disadvantage characterize RA patients with fibromyalgia. *The Journal of Rheumatology*.

[18] Naranjo A., Ojeda S., Francisco F., Erausquin C., Rúa-Figueroa I., Rodríguez-Lozano C. (2002). Fibromyalgia in patients with rheumatoid arthritis is associated with higher scores of disability. *Annals of the Rheumatic Diseases*.

[19] Edwards R. R., Wasan A. D., Bingham C. O. (2009). Enhanced reactivity to pain in patients with rheumatoid arthritis. *Arthritis Research and Therapy*.

[20] Arendt-Nielsen L., Graven-Nielsen T. (2011). Translational musculoskeletal pain research. *Best Practice & Research: Clinical Rheumatology*.

[21] Lemming D., Graven-Nielsen T., Sörensen J., Arendt-Nielsen L., Gerdle B. (2012). Widespread pain hypersensitivity and facilitated temporal summation of deep tissue pain in whiplash associated disorder: an explorative study of women. *Journal of Rehabilitation Medicine*.

[22] Studenic P., Radner H., Smolen J. S., Aletaha D. (2012). Discrepancies between patients and physicians in their perceptions of rheumatoid arthritis disease activity. *Arthritis and Rheumatism*.

[23] Navarro-Sarabia F., Ruiz-Montesinos D., Hernandez B. (2009). DAS-28-based EULAR response and HAQ improvement in rheumatoid arthritis patients switching between TNF antagonists. *BMC Musculoskeletal Disorders*.

[24] Whittle S. L., Colebatch A. N., Buchbinder R. (2012). Multinational evidence-based recommendations for pain management by pharmacotherapy in inflammatory arthritis: integrating systematic literature research and expert opinion of a broad panel of rheumatologists in the 3e initiative. *Rheumatology*.

